# Author Response: Relationship of Choroidal Vasculature and Choriocapillaris Flow With Alterations of Salivary α-Amylase Patterns in Central Serous Chorioretinopathy

**DOI:** 10.1167/iovs.63.6.10

**Published:** 2022-06-09

**Authors:** Fabio Scarinci, Francesca Romana Patacchioli, Eliana Costanzo, Mariacristina Parravano

**Affiliations:** 1 IRCCS – Fondazione Bietti, Rome, Italy. E-mail: fabioscarinci@gmail.com.

We thank Menean et al.^1^ for giving us the opportunity to further discuss important methodological aspects carefully observed in our study regarding the reliable assessment of salivary α-amylase (α-AMY) as a marker of autonomic nervous system (ANS) activity in acute central serous chorioretinopathy (CSC).

We would like to start by recalling the conclusions of our paper^2^: “As a whole, we believe that the dysregulation of the functional chronobiology of the ANS should be included among the trigger factors of some of the acute CSC features.” Objectively, we do not see substantial differences in what Menean et al.^1^ say in the first paragraph of their letter.

That said, we would like to clarify a main possible issue of misunderstanding from which a large proportion of the comments made by Menean et al.^1^ might arise. It is interesting to speculate that organisms not only regulate their physiological functioning in response to acute and chronic challenges, but also rely on biological rhythms to continuously adapt to the environment. Thus, we think that alterations in biological rhythms might impair health and well-being, just as different stressor exposures can, obviously with mechanisms not always overlapping.

We are very confident that salivary α-AMY (and cortisol) measures might be helpful for describing the mechanisms by which several challenges potentially contribute to the pathogenesis and outcomes of stress-related diseases in predisposed individuals.^3^^–^^5^ For this purpose, it has been previously shown that different salivary biomarkers are indicators of stress-related hypothalamic–pituitary–adrenocortical axis and ANS activities, in which the activation of the stress response is induced by contingent and variable sources of external stimuli, such as hypoxia induced by hypobaric chamber challenge or vertigo induced by caloric testing.^6^^,^^7^

In contrast, both of the studies we have been invited to discuss here describe the hypothalamic–pituitary–adrenocortical axis and ANS activity in resting conditions under a relatively fixed source of stimulation, such as internal rhythms that regulate the daily periodicity of the systems, quite independently of feedback circuits.^8^ Here, it is important to remember that our studies were carried out under resting conditions, verified through diaries and compliance checks on the study participants regarding saliva collection.

Menean et al.^1^ had doubts regarding the potential influence of the parasympathetic drive on salivary flow regulation and, therefore, the accuracy of our salivary α-AMY measures, quoting a study that pointed out a possible decrease in the salivary flow rate under the stress response as a confounding factor.^9^

To comment on this point of discussion, first, we cannot omit a study by researchers of excellence in the field of salivary stress biomarkers, which reported that increased salivary α-AMY production under conditions of stress was distinct from changes in the salivary flow rate.^10^ The authors showed that the response patterns of salivary α-AMY during controlled laboratory stress were consistent, regardless of whether the AMY concentration (U/mL) or the AMY output (U/min) was examined.

However, to overcome any bias on salivary α-AMY measures owing to salivary flow, we must point out that, in our studies, the salivary flow rate was in fact calculated.^9^^,^^11^ As indicated in the methods section, the unstimulated salivary flow rate (mL/min^−1^) was determined in the study population by dividing the volume of saliva by the collection time. Under basal conditions, the rate of saliva production was 0.5 mL/min^−1^. The salivary flow rate of valid samples should not be less than 0.1 mL/min^−1^.^12^^,^^13^

Thus, we do not believe that salivary flow cannot be controlled.

Under “unstimulated” resting conditions, our protocol provided a multiple-timepoint assessment of salivary α-AMY production characterizing the activity of ANS in acute CSC compared with healthy matched controls.^2^^,^^14^ In our opinion, not including α-AMY levels within the first hour of waking does not represent poor accuracy, but rather the choice to emphasize changes in the diurnal variation trend rather than those closer to awakening. Therefore, we intentionally decided not to insert the salivary α-AMY values at 0, 30, and 60 minutes after waking that were collected and reported in a previous study.^14^

We already speculatively discussed in a review^15^ and explicitly reported in our study^2^ that changes in diurnal salivary α-AMY production (diurnal percentage variation, ANS associated) observed in acute CSC-susceptible individuals could be attributed to physiological system dysregulation (dysfunction or imbalance), allowing us to infer a causal link between ANS derangement (imbalance) and choroidal acute CSC features.

In addition, we would like to emphasize that the study population was not exposed to any type of stress, acute or chronic. Moreover, in our studies, only subjects with acute CSC were involved.

Our previous work assessed circadian patterns of basal salivary α-AMY secretion in acute CSC^14^; several composite variables (diurnal trends of the biomarker and area under the curve with respect to the ground) were derived from the values measured at the repeated saliva collection times, showing higher levels of α-AMY in the CSC group than in controls, which was peculiarly detectable in the second part of the day. Conversely, in the study published in *Investigative* *Ophthalmology and* *&* *Visual* *Science*,^2^ raw biomarker data were used to estimate (1) the area under the curve with respect to the ground, showing a significant increase of α-AMY production from 8:00 to 20:00 h. Furthermore, (2) the relative percentage variation between evening and morning production of α-AMY was estimated, showing a decrease close to 30% in the expected increase in the evening percentage of α-AMY production in CSC subjects compared with the control group. In our opinion, the reported results add different observation points to deepen the knowledge of acute CSC and its involvement with ASN, a very complex system, using the measurement of α-AMY as a surrogate marker of sympathetic activity.^2^^,^^14^^–^^17^

We are interested in reading the in vitro study cited by Menean et al.^1^ concerning the implications of the differential expression of receptors for glucocorticoid hormones in rat ocular tissues in an experimental model of hypothalamic–pituitary–adrenocortical axis downregulation.^18^ However, from the currently available literature, we do not believe it is possible to establish or even exclude a link between salivary α-AMY changes and glucocorticoid receptors (both mineralocorticoid and glucocorticoid receptor) in humans with acute/chronic CSC.^2^^,^^15^^–^^17^^,^^19^^,^^20^
